# Remineralizing Treatments for Dental Erosion and Sensitivity in Patients Suffering from Gastroesophageal Reflux Disease (GERD): Randomized Clinical Trial

**DOI:** 10.3390/jcm14103525

**Published:** 2025-05-18

**Authors:** Andrea Scribante, Alessia Pardo, Maurizio Pascadopoli, Federico Biagi, Annalisa Schiepatti, Valentina Giammona, Marco Vecchio, Christian Alquati, Gioia Giada Modica, Cinzia Casu, Andrea Butera

**Affiliations:** 1 Unit of Dental Hygiene, Section of Dentistry, Department of Clinical, Surgical, Diagnostic and Pediatric Sciences, University of Pavia, 27100 Pavia, Italy; andrea.scribante@unipv.it (A.S.); marco.vecchio01@universitadipavia.it (M.V.); christian.alquati01@universitadipavia.it (C.A.); gioiagiada.modica@unipv.it (G.G.M.); 2 Unit of Orthodontics and Pediatric Dentistry, Section of Dentistry, Department of Clinical, Surgical, Diagnostic and Pediatric Sciences, University of Pavia, 27100 Pavia, Italy; maurizio.pascadopoli01@universitadipavia.it (M.P.); valentina.giammona02@universitadipavia.it (V.G.); 3 Section of Dentistry and Maxillofacial Surgery, Department of Surgical Sciences, Pediatrics and Gynecology, University of Verona, 37124 Verona, Italy; 4 Department of Internal Medicine and Therapeutics, University of Pavia, 27100 Pavia, Italy; federico.biagi@unipv.it (F.B.); annalisa.schiepatti@unipv.it (A.S.); 5 Gastroenterology Unit, Pavia Institute, Istituti Clinici Scientifici Maugeri IRCCS, 27100 Pavia, Italy; 6 Oral Biotechnology Laboratory (OBL), Department of Molecular Biology, University of Cagliari, 09121 Cagliari, Italy; cinzia.casu2@unica.it

**Keywords:** gastroesophageal reflux, tooth erosion, tooth remineralization, hydroxyapatite

## Abstract

**Background:** Gastroesophageal reflux disease (GERD) is a chronic condition that causes an abrupt decrease in salivary pH in the oral cavity, which can lead to demineralization, erosion, hypersensitivity, functional impairment, and possibly fracture of dental elements. The aim of this clinical study is to compare two types of treatment in patients with dental erosion diagnosed with gastroesophageal reflux. **Methods:** Thirty patients were enrolled in this randomized clinical trial. Each patient underwent clinical evaluation and esophageal pH measurement, in order to diagnose GERD. After an initial examination and assessment by an experienced dentist, the Trial group (15 patients) was assigned to home treatment with a zinc hydroxyapatite-based toothpaste and a hydroxyapatite-based paste, while the Control group (15 patients) was assigned to home treatment with zinc hydroxyapatite-based toothpaste only. The following indices were measured: Basic Erosive Wear Examination Index (BEWE); Schiff Air Index (SAI); Plaque Index (PI); and Bleeding Score (BS). Each index was assessed at T0 during the first visit, one month (T1), three months (T2), six months (T3), nine months (T4), and 12 months (T5). The Kolmogorov–Smirnov test was used to analyze the normality of the data, while Friedman’s test followed by Dunn’s post hoc test were used to compare the two groups (significance threshold: *p* < 0.05). **Results**: The results showed no statistically significant change in the BEWE and SAI indexes (*p* > 0.05). However, an improvement in dentin sensitivity and BS was observed. Plaque control also improved. **Conclusions**: The results of this study indicate that the additional hydroxyapatite paste did not significantly improve the outcomes of the study in respect to hydroxyapatite toothpaste alone. However, there was an improvement in the oral health of GERD patients using hydroxyapatite-based remineralizing treatment in terms of oral and periodontal indices calculated.

## 1. Introduction

Dental enamel consists of 96–97% inorganic material, mainly calcium phosphate organized into hydroxyapatite crystals, and 2–3% organic material. The prisms in the enamel provide high mechanical strength. However, once the tooth has erupted into the oral cavity, the enamel is no longer able to repair itself [1].

Oral pH is normally between 6.5 and 7.5, but a drop to 5.5 causes an increase in the solubility of hydroxyapatite, leading to tooth erosion. The latter is defined as the irreversible loss of dental hard tissue without the involvement of microorganisms [2].

The cause of erosion is usually the presence of acidic agents in the oral cavity, and the solubility constant of hydroxyapatite varies according to the specific properties of each acid. In fact, dental erosion can be caused by both intrinsic and extrinsic substances [3,4]. Extrinsic causes include acidic foods (e.g.: citrus fruits, wine, salad dressing), carbonated drinks, acidic drugs, sugary snacks, frequency of sugar intake, and environmental exposure to acidic substances [5,6]

On the other hand, anorexia nervosa, bulimia, and gastrointestinal disorders such as gastroesophageal reflux disease (GERD), which cause frequent regurgitation, are intrinsic causes because highly acidic gastric substances are forced into the oral cavity [7,8].

GERD is a chronic disease [9] that can present with symptoms such as abdominal pain, regurgitation, dysphagia, cough, hoarseness, and chest pain. Extra-esophageal manifestations are common but often go undiagnosed. The essential test to diagnose gastroesophageal reflux is esophageal pH measurement. The probe records the esophageal pH over a period of 24–48 h. At the end of the monitoring, the data are analyzed to determine the number, duration, and percentage of times the pH is below 4, the threshold for acid reflux [9,10,11,12].

Untreated GERD can lead to hypersensitivity due to tooth erosion, functional impairment, and possibly tooth fracture [13]. In addition, side effects on soft tissues were reported, such as gingival and palatal erythema, glossitis, gingival ulcer, and erythema of the floor of the mouth; additionally, the occurrence of gingivitis appears to be very high, affecting almost 67% of cases [14].

To address the problem of tooth demineralization, different molecules have been introduced over time: from fluoride [15,16,17,18] to casein [19,20,21] to apatite hydroxides [22,23]. These compounds have shown encouraging results both on demineralized areas [24,25] and on initial caries [26,27] or teeth with mineralization defects [28,29]. Considering the results of previous reports, hydroxyapatite has shown a remineralizing activity and enhanced the reduction of hypersensitivity, which is the first step in remineralization [17,23].

Therefore, the aim of this study is to compare the effectiveness of two remineralizing treatments in patients with GERD and dental erosion by evaluating dental and periodontal parameters. In the Trial group, participants used both a hydroxyapatite-based toothpaste and a hydroxyapatite-based paste as part of their home oral care routine, whereas in the Control group only the toothpaste was used. This design aimed to assess the potential additional benefits provided by the paste when used in combination with the toothpaste in terms of a reduction in dental sensitivity and erosion.

The null hypothesis is that there are no differences between the two treatments at the end of the study.

## 2. Materials and Methods

### 2.1. Trial Designs

This study was a randomized clinical trial with a 1:1 allocation ratio. Approval from the Unit Internal Review Board was obtained (ID: 2022-0126). The protocol was registered on the clinicaltrials.gov platform (NCT number: NCT05371717)

### 2.2. Participants

The study was conducted at the Dental Hygiene Unit, Dentistry Section, Department of Clinical, Surgical, Diagnostic and Pediatrics Sciences, University of Pavia (Pavia, Italy), and included patients with GERD treated at the Gastroenterology Unit of the ICS Maugeri of Pavia, who were referred to the Dental Hygiene Unit to start a treatment pathway. The eligibility criteria are listed in Table 1.

### 2.3. Interventions and Outcomes

At the first visit (T0), patients underwent a thorough medical history and periodontal assessment by an experienced clinician. The clinician was calibrated after conducting an evaluation of 5 patients, who were not included in the study but underwent the collection of the same indices as those in this report. The evaluations were performed twice in an interval of 14 days. Patients were given information on correct home oral hygiene maneuvers according to their individual level of oral hygiene, dental element morphology and position, age, and manual dexterity.

The following indices were then evaluated:

- The Basic Erosive Wear Examination Index (BEWE) presents criteria for evaluation based on 4 scores (0 to 3) assigned according to the amount of hard tissue lost and the ability to visually identify it [30];
- The Schiff Air Index (SAI) quantifies the pain of dental hypersensitivity in response to the evaporative stimulus (no pain, response to stimulation, response to stimulation and removal, accentuated pain response) by assigning a score ranging from 0 to 3 [31];
- The Plaque Index (PI) is recorded during the clinical periodontal examination at 4 sites for each tooth element present, using circumferential probing with a manual periodontal probe [32];
- The Bleeding Score (BS) is based on the amount of bleeding after periodontal probing; the index ranges from no bleeding to profuse and copious bleeding [33].

After the evaluation of the indices, the patients underwent a professional oral hygiene session performed by the operator; the instruments used consisted of a piezoelectric instrument (Multipiezo, Mectron S.p.a, Carasco, Italy) and Gracey curettes (Hu-Friedy, Chicago, IL, USA).

Patients were randomized into two groups:

- in the Trial group, a zinc hydroxyapatite-based toothpaste (Biorepair Plus Total Protection, Biorepair^®^, Coswell S.p.A., Funo di Argelato, Italy) treatment was applied with a toothbrush (manual or electric) to the buccal and lingual surfaces of the dental elements twice daily for the duration of the study. In addition, a hydroxyapatite-based paste was applied (Biorepair Plus Enamel Repair Intensive Treatment, Biorepair^®^ Coswell, S.p.A., Funo di Argelato, Italy). After the oral hygiene procedures, the treatment was applied to the inner surface of a bite guard, which was then placed in the patient’s mouth. The patients were instructed to close their teeth naturally, allowing the product to act for 10 min. The treatment was repeated for 7 days per month for the entire length of the study.
- Zinc hydroxyapatite-based toothpaste (Biorepair Plus Total Protection) was applied in the Control group as indicated for the Trial group. No additional paste or treatment was applied.

The compositions of the two tested products are shown in Table 2.

The same procedures were performed at each session, i.e., after one month (T1), after 3 months (T2), after 6 months (T3), after 9 months (T4) and after 12 months (T5) from baseline. Patient adherence to the home protocol was assessed by asking at each visit whether they were continuing with the application of the products tested.

### 2.4. Sample Size

The sample size was calculated considering the Schiff Air Index as the primary outcome. The study by Pepelassi et al. [34] was used for the calculation, considering data on the Schiff Air Index at the end of the treatment between the two study groups, and considering a higher efficacy of the paste plus toothpaste versus toothpaste alone in reducing dental sensitivity, with an expected mean of 0.41, an expected mean difference of 0.56, and a standard deviation of 0.55. Setting an alpha error = 0.05 and power = 80%, 15 patients per group were required, with a total of 30 patients for this study.

### 2.5. Randomization and Blinding

A block randomization table was used for patient allocation. A random sequence was generated by the data analyst, considering a permuted block of 30 participants in total. One operator (A.P.), who had not been involved in the previous procedures, enrolled and administered the professional verbal procedures and recorded the results. Using pre-prepared, sequentially numbered, opaque, sealed envelopes (SNOSEs), an assistant allocated participants to their respective groups. The data analyst was unaware of the allocation and results. The home oral hygiene products were concealed.

### 2.6. Statistical Analysis

Data were statistically analyzed with R software (R version 3.1.3 R Development Core Team, R Foundation for Statistical Computing, Vienna, Austria), calculating descriptive statistics for each variable, including: mean, standard deviation, median, minimum and maximum values, measured for each group. The normality of the distributions was assessed using the Kolmogorov–Smirnov tests. For the demographic data, the Mann–Whitney test was performed. For intragroup comparisons, Friedman’s test was then applied, followed by Dunn’s post hoc test for multiple comparisons. For inter-group comparisons, the Kruskal–Wallis test and Dunn tests were applied. For all tests, significance was set at *p* < 0.05. For all tests, significance was set at *p* < 0.05.

## 3. Results

### 3.1. Participants Flow and Baseline Data

**Figure 1:**
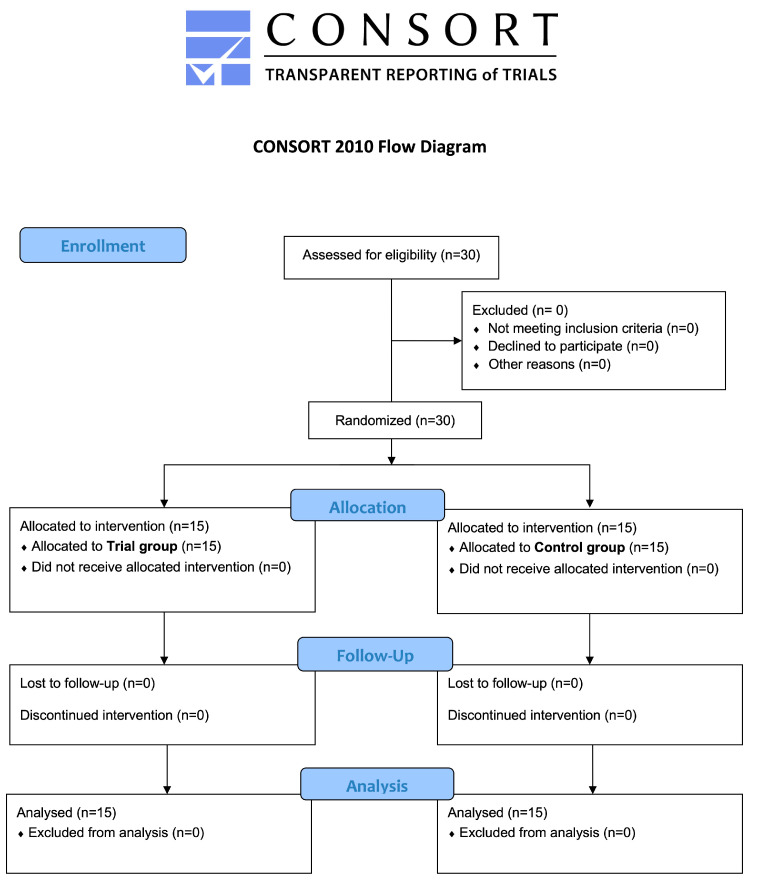
enrollment, allocation, follow-up, and analysis.

Recruitment continued until a total of 30 patients were enrolled. Once enrolled, patients who agreed to participate in the study were divided into two groups and they all ended the study. The flowchart of the study is shown in Figure 1. Regarding demographic data, the mean age of participants was 52.0 ± 6.7 in the Trial group and 50.1 ± 13.6 in the Control group, with no significant difference according to the Mann–Whitney test (*p* = 0.7048). Regarding gender distribution, there were 21 females (70%) and 9 males (30%) in total (*p* = 0.0041*). In the Trial group, 6 (20%) males and 9 (30%) females were enrolled, while in the Control group 3 (10%) males and 12 (40%) females were enrolled. The comparison between the Control and Trial group was not significant for males (*p* = 0.427) and females (*p* = 0.427).

To denote both within- and between-group comparisons at the same time, a letter-based comparison was adopted, meaning that groups that share at least one identical significant letter are not considered significantly different [35].

### 3.2. BEWE

As shown in Table 3, there was no significant within-group change (*p* > 0.05) between the Control and Experimental groups. Furthermore, even within each group, there is no evidence of any significant change between time T0 and time T5 (*p* > 0.05).

### 3.3. Schiff Air Index

Regarding the SAI (Table 4), there were not statistically significant within-group differences in the Control and Test groups (*p* > 0.05). The only significant between-group differences were Control T3 vs Trial T0 (*p* = 0.005), Control T4 vs Trial T0 (*p* = 0.01), and Control T5 vs Trial T0 (*p* = 0.01).

### 3.4. Bleeding Score

Regarding the BS index (Table 5), no significant within-group difference was observed in the Control group, while in the Trial the only significant comparisons found were Trial T0 vs Trial T4 (*p* = 0.042) and Trial T0 vs Trial T5 (*p* = 0.042). Between-group comparisons were not significant (*p* > 0.05). In both cases, there was a reduction in gingival bleeding levels.

### 3.5. Plaque Index

In terms of PI (Table 6), the within-group analysis revealed the following significant differences in the Control group: T0 vs T3 (*p* = 0.014); T0 vs T4 (*p* = 0.004); and T0 vs T5 (*p* = 0.005). No significant within-group differences were found in the Trial group (*p* > 0.05) and no between-group differences were found (*p* > 0.05).

### 3.6. Ph-Metry

The data concerning the pH-metry of patients with GERD are presented in Table 7. At baseline, there was no significant difference between the two groups (*p* > 0.1)

## 4. Discussion

Acid reflux is a condition in which the acids in the stomach are continually regurgitated into the esophagus and then into the mouth. GERD affects an average of 10–20% of the world’s population [36]. GERD was first associated with dental erosion in 1937 by Bargen and Austin [37]. Due to the decrease in pH caused by acid reflux (pH< 5.5), dental erosion, i.e., initial damage to the enamel prisms and loss of tooth substance, can occur if the ideal pH of the oral cavity is not restored [38]. The palatal side of the upper incisors is the first to be affected by the acid attack of gastroesophageal reflux; if the situation persists, the occlusal surfaces of the teeth in both arches will be eroded [39]. Additionally, the occlusal and buccal surfaces of the lower arches are the last to be affected by tooth erosion due to the natural shield provided by the tongue [39]. When the oral pH approaches neutrality, saliva promotes remineralization, allowing the reabsorption of minerals lost from the enamel.

There are many articles in the literature that discuss products studied to remineralize eroded surfaces. This is a topic that stimulates research interest and requires further clinical studies. In fact, among the articles available in the literature, most are in vitro studies, while a few clinical studies are available. Moreover, clinical studies that explore the remineralization of dental surfaces in patients affected by GERD are missing. Therefore, the objective of our clinical study was to test a treatment consisting of a toothpaste based on zinc-hydroxyapatite, along with a paste with the same composition, compared to a treatment consisting only of a toothpaste containing zinc-hydroxyapatite.

Among the different ways of promoting remineralization, one of the most important and most studied is fluoride. It facilitates remineralization by promoting the formation of fluorapatite, a crystalline structure that is more resistant to acid attack than hydroxyapatite [40,41].

In addition to fluoride, calcium and phosphate, in combination or alone, play a crucial role in the remineralization process. Their presence in saliva and in therapeutic formulations (such as amorphous calcium phosphate toothpastes) is crucial for the remineralization of damaged enamel [42]. Another option is the use of casein calcium phosphopeptide-amorphous calcium phosphate (CPP-ACP), which is a protein complex derived from casein that acts as a calcium and phosphate reservoir in response to a drop in pH, stabilizing amorphous calcium phosphate by facilitating its availability on the tooth surface, thus promoting the remineralization process [43]. Several in vitro studies [44,45] have compared the efficacy of fluoride with other compounds, such as calcium glycerophosphate (CaGP) and Casein Phosphopeptide—Amorphous Calcium Fluoride (CPP-ACFP). In both cases, the best clinical outcomes were observed with fluoride-based products, as assessed through microscopy and surface composition analysis. The remineralization of dental surfaces, even when erosion is extended to the dentin, was more effective with fluoride, either alone or in combination with other components, such as amine fluoride.

Remineralization of dental tissues using biomimetic hydroxyapatite (HAp) is one of the latest innovations in preventive and restorative dentistry [22,25]. Biomimetic hydroxyapatite works by releasing calcium and phosphate ions that integrate into the matrix of demineralized enamel, facilitating remineralization at both the surface and sub-surface levels. This process occurs through the nucleation of hydroxyapatite crystals, which are deposited on the enamel lesion, forming a new mineral layer that mimics the natural structure [46]. In addition, HAp can penetrate and seal microfractures in enamel and dentin, facilitating remineralization and restoring the crystalline structure of damaged enamel, thereby contributing to the reduction of tooth sensitivity [47,48].

In this study, the null hypothesis was partially rejected as differences were found between the two groups even if the clinical outcomes were very similar. In fact, patients treated with zinc-hydroxyapatite-based toothpaste in combination with zinc-hydroxyapatite-based paste presented a noticeable decrease in sensitivity, analyzed by the SAI, within the first 3 months, while case patients treated with the toothpaste alone experienced a decrease within 6 months. No significant between-group difference was found for the BEWE index, even though a clinical reduction within groups was observed. Statistically significant differences were observed in the Trial group for the BS in the comparisons T0 vs T4 and T0 vs T5, while no significant difference was found in the Control group as well as no significant inter-group comparisons. The PI, on the other hand, showed a significant improvement only in the Control group for the comparisons T0 vs T3, T0 vs T4, and T0 vs T5, with no significant intragroup comparisons in the Trial group and no significant inter-group comparisons.

However, it should be noted that, in the specific type of patients recruited for the study, the efficacy of the treatment could be lower due to the constant presence of the acidic environment of the oral cavity, which prevents the restoration of a suitable pH for remineralization [2]. Therefore, additional treatments may be required. It is also important to differentiate the pH to which teeth are exposed when assessing the effectiveness of hydroxyapatite-based treatments. [49].

In this study, where the oral cavity pH often reached values less than or equal to 4, there was neither an improvement nor a worsening in the BEWE index, indicating that there was no clinically detectable remineralization or further demineralization.

Additionally, this report also evaluated gingival inflammation. A clinical decrease was observed in the BS and PI, which improved in both the Trial and the Control group.

Concerning the use of HAp, Kani and co-workers [47] evaluated the efficacy of a biomimetic hydroxyapatite toothpaste in subjects with tooth sensitivity. The results showed a significant reduction in sensitivity after four weeks of use, which was attributed to HAp’s ability to occlude exposed dentinal tubules. The study conducted by Kani et al. agrees with this study, as both the Trial Group and the Control group showed a reduction in SAI, although this was not statistically significant. This improvement could be due to the deposition of HAp inside dentinal tubules, leading to their obliteration and, consequently, an improvement in sensitivity.

Scribante et al. [50] investigated the efficacy of two remineralizing toothpastes: one based on zinc carbonate hydroxyapatite and the other containing magnesium strontium carbonate hydroxyapatite conjugated with chitosan. This six-month study considered various parameters (DIAGNOdent Pen Index, BEWE Index, Plaque Index, Bleeding Score, Schiff Air Index, and International Caries Detection and Assessment System—ICDAS). The results showed a reduction in the DIAGNOdent Pen score in the Trial group after one month, along with a decrease in the Plaque Index, while the other indices remained unchanged. This suggests that zinc-hydroxyapatite promotes greater remineralization in the short term.

In accordance with these results, this study showed a statistically significant reduction in PI in the Control group at T5 compared to T0. In the Trial group, there was also a clinically appreciable reduction in PI. In addition to PI, both BS and SAI showed significant improvement in the Trial group. These findings indicate that HAp- zinc products, such as the toothpaste and the paste, have contributed to an improvement in mineralization, even if the analyzed time period was quite short.

Conversely, a study by Jung et al. [51] aimed at testing whether HAp-containing formulations could reduce dental erosion and abrasion yielded unfavorable results. Two HAp-based pastes with fluoride, two without fluoride, and a mouthwash were tested. Only the fluoride and stannous-based mouthwash prevented dental surface erosion, while none of the HAp-containing products were effective in protecting against it. Furthermore, tooth brushing led to increased tissue loss in all cases except for the fluoride/stannous mouthwash group.

In this study, the amount of hard tissue loss was not assessed; therefore, a direct comparison with the abovementioned in vitro study was not possible. However, it can be hypothesized that HAp enabled remineralization and occlusion of the dentinal tubules due to the fact that an improvement in SAI was noted. Further studies should consider a comparison between the HAp molecule and fluoride-containing products to confirm or disprove the results here obtained.

Due to the lack of clinical studies assessing the remineralizing potential of these products in patients with GERD, conditions associated with demineralization, such as molar incisor hypomineralization (MIH) and white spot lesions (WSLs), were analyzed.

Both Orilisi G. and Esparza-Villalpando [52,53] analyzed the effectiveness of different types of toothpaste (hydroxyapatite with fluoride ions, sodium monofluorophosphate with arginine, sodium fluoride with enzymes, and casein phosphopeptide-amorphous calcium phosphate) on teeth affected by white spot lesions (WSLs), which are the early on-set stages of non-cavitated carious lesions characterized by a chalky-white appearance due to hydroxyapatite dissolution [54]. Various parameters, such as surface micromorphology analyzed by scanning electron microscopy (SEM), chemical/elemental composition (RMS and EDS), Vickers microhardness (VMH), and fluorescence units (FUs), were evaluated, showing an improvement in all cases. This study thus confirms the efficacy of hydroxyapatite, even when combined with fluoride ions; however, it does not demonstrate its superiority over other molecules, but rather its non-inferiority.

The studies mentioned in this discussion consider various molecules that promote the remineralization of enamel and dentin erosion-affected elements, while indicating the non-inferiority of HAp compared to other molecules. As observed in the clinical study conducted on patients with GERD, a partial remineralization of dental structures occurred, highlighting the remineralizing potential of HAp in various clinical conditions. These studies encourage further clinical research to test and compare different molecules, such as fluoride-hydroxyapatite or CPP-ACFP, in patients with GERD. Since these are all clinical studies conducted on patients, patients’ compliance with the study protocol should be taken into consideration. 

Based on the analyzed studies, we can conclude that HAp is a highly effective molecule in the remineralization of dental elements affected by erosion, regardless of its cause, whether due to GERD, WSL, MIH, or erosion induced in in vitro studies. However, its superiority over other molecules cannot be established, as fluoride, CPP-ACFP, sodium monofluorophosphate with arginine, and sodium fluoride with enzymes have also demonstrated significant results in the mineralization of dental structures.

Furthermore, this study suggests that HAp, in a toothpaste formulation combined with paste, does not show statistically superior effects compared to the use of toothpaste alone in terms of dental sensitivity and erosion. This finding may be explained by the substance reaching its maximum effectiveness at a concentration achievable with toothpaste alone or by the inability to remineralize severely demineralized structures solely through chemical compounds, without the intervention of a restoration to fully reconstruct the dental anatomy. Further studies could consider using the additional paste as an adjunct to the daily home application of toothpaste and not only for 10 days a month.

Among the limitations of the study, the health status of patients with GERD during the study was not investigated and therefore it is not known whether there was a worsening of the clinical condition of the patients. Additionally, the potential different compliance among patients was not investigated.

In the future, other instruments, such as DIAGNOdent or intraoral scanners, could be used to evaluate the amount of enamel condition before and during remineralizing treatments. Finally, it would be useful to also evaluate the patient’s perspective by testing satisfaction after the treatment and their perception of oral health-related quality of life. 

## 5. Conclusions

The results of the study show that there were statistically significant improvements in the Control group regarding PI. There was a statistically significant reduction in BS in the Trial group and a reduction in SAI, although it was not significant. However, at the end of the therapy, no statistically significant differences were observed between the Trial and Control groups. Therefore, the additional hydroxyapatite-based paste did not improve the clinical performances of the hydroxyapatite-based toothpaste administered in both the study groups. In both cases, there was no worsening of the erosion conditions, indicating that HAp acted as a protective factor, preventing dental tissues from further demineralization in patients with GERD and exerting a proactive action in the whole oral cavity. Gastroenterologists could play a key role in initiating oral health prevention procedures by recommending remineralizing products to their patients.

## Figures and Tables

**Table 1:** Inclusion and exclusion criteria of the study.

**Inclusion Criteria**	Patients with dental erosion Patients with diagnosed gastroesophageal reflux No proton pump inhibitor medication prior to PH examination
**Exclusion Criteria**	Absence of dental erosion Patients with psychiatric or neurological problems Patients under 18 years of age Patients with systemic diseases Pregnant or lactating patients Patients with poor compliance

**Table 2:** Chemical composition of toothpastes used.

Zinc hydroxyapatite-based toothpaste (Biorepair Plus Total Protection)	Aqua, Zinc Hydroxyapatite (microRepair^®^) 20%, Glycerin, Hydrated Silica, Sorbitol, Silica, Aroma, Cellulose Gum, Sodium Myristoyl Sarcosinate, Sodium Methyl Cocoyl Taurate, Citric Acid, Tetrapotassium Pyrophosphate, Zinc PCA 13%, Sodium Saccharin, Phenoxyethanol, Benzyl Alcohol, Sodium Benzoate
Zinc-hydroxyapatite-based paste (Biorepair Plus Enamel Repair Intensive Treatment)	Aqua, Zinc Hydroxyapatite (microRepair^®^), Hydrated Silica, Silica, Sodium Myristoyl Sarcosinate, Sodium Methyl Cocoyl Taurate, Sodium Bicarbonate, Aroma, Sodium Saccharin, Phenoxyethanol, Benzyl Alcohol, Sodium Benzoate, Citric Acid, Menthol

**Table 3:** BEWE index. *: means with same letters are not significantly different (*p* > 0.05).

		Mean	SD	Min	Mdn	Max	Significance *
Control	T0	1.33	0.49	1.00	1.00	2.00	A
	T1	1.27	0.46	1.00	1.00	2.00	A
	T2	1.20	0.41	1.00	1.00	2.00	A
	T3	1.13	0.35	1.00	1.00	2.00	A
	T4	1.13	0.35	1.00	1.00	2.00	A
	T5	1.13	0.35	1.00	1.00	2.00	A
Trial	T0	1.40	0.51	1.00	1.00	2.00	A
	T1	1.33	0.49	1.00	1.00	2.00	A
	T2	1.13	0.35	1.00	1.00	2.00	A
	T3	1.00	0.00	1.00	1.00	1.00	A
	T4	1.00	0.00	1.00	1.00	1.00	A
	T5	1.00	0.00	1.00	1.00	1.00	A

**Table 4:** SAI scores. *: means with same letters are not significantly different (*p* > 0.05).

		Mean	SD	Min	Mdn	Max	Significance *
Control	T0	1.07	0,80	0.00	1.00	3.00	A,B
	T1	0.93	0.70	0.00	1.00	2.00	A,B
	T2	0.67	0.72	0.00	1.00	2.00	A,B
	T3	0.40	0.63	0.00	0.00	2.00	B
	T4	0.47	0.64	0.00	0.00	2.00	B
	T5	0.47	0.64	0.00	0.00	2.00	B
Trial	T0	1.53	0.92	0.00	2.00	3.00	A
	T1	1.20	1.01	0.00	1.00	3.00	A,B
	T2	1.00	0.85	0.00	1.00	3.00	A,B
	T3	0.93	0.88	0.00	1.00	3.00	A,B
	T4	0.93	0.88	0.00	1.00	3.00	A,B
	T5	0.93	0.92	0.00	1.00	3.00	A,B

**Table 5:** Bleeding Score values. *: means with same letters are not significantly different (*p* > 0.05).

		Mean	SD	Min	Mdn	Max	Significance *
Control	T0	0.45	0.20	0.15	0.50	1.00	A,B
	T1	0.42	0.16	0.12	0.50	0.67	A,B
	T2	0.34	0.18	0.10	0.35	0.67	A,B
	T3	0.35	0.20	0.10	0.30	0.67	A,B
	T4	0.31	0.18	0.10	0.30	0.67	A,B
	T5	0.31	0.18	0.10	0.30	0.67	A,B
Trial	T0	0.46	0.20	0.22	0.50	1.00	A
	T1	0.36	0.20	0.11	0.32	0.93	A,B
	T2	0.35	0.21	0.10	0.30	0.95	A,B
	T3	0.34	0.21	0.10	0.30	0.90	A,B
	T4	0.31	0.21	0.08	0.30	0.90	B
	T5	0.33	0.21	0.08	0.30	0.90	B

**Table 6:** Plaque Index. *: means with same letters are not significantly different (*p* > 0.05).

		Mean	SD	Min	Mdn	Max	Significance *
Control	T0	0.65	0.19	0.30	0.62	1.00	A
	T1	0.59	0.20	0.35	0.50	1.00	A,B
	T2	0.53	0.20	0.30	0.50	1.00	A,B
	T3	0.41	0.12	0.17	0.45	0.56	B
	T4	0.39	0.12	0.17	0.40	0.56	B
	T5	0.39	0.13	0.17	0.45	0.56	B
Trial	T0	0.57	0.22	0.25	0.50	1.00	A,B
	T1	0.45	0.22	0.20	0.50	1.00	A,B
	T2	0.52	0.22	0.20	0.50	1.00	A,B
	T3	0.49	0.19	0.20	0.50	1.00	A,B
	T4	0.47	0.19	0.20	0.50	1.00	A,B
	T5	0.47	0.20	0.20	0.50	1.00	A,B

**Table 7:** pH-metry scores among the groups. *: means with same letters are not significantly different (*p* > 0.05).

	Mean	SD	Min	Mdn	Max	Significance *
Control	0.16	0.40	0.00	0.00	1.00	A
Trial	0.33	0.51	0.00	0.00	1.00	A

## Data Availability

Data are available upon reasonable request to the corresponding authors.
